# Supplementary material for: From sequence to enzyme mechanism using multi-label machine learning
Source: BMC Bioinformatics. 2014 May 19;15:150. doi: 10.1186/1471-2105-15-150 (PMC4229970; doi:10.1186/1471-2105-15-150)
Supplement: Additional file 2 — Java code of ml2db. Additional file ml2db_code.tar.gz contains the Java source code to run the multi-label machine learning experiments and save the results to database. The code’s Javadoc is included. [file 1471-2105-15-150-S2.zip › additional file 2/ml2db/utils/doc/overview-tree.html]

Class Hierarchy


JavaScript is disabled on your browser.


- Overview
- Package
- Class
- Use
- Tree
- Deprecated
- Index
- Help

- Prev
- Next

- Frames
- No Frames

- All Classes

# Hierarchy For All Packages

Package Hierarchies:

- cern.colt,
- cern.colt.function,
- cern.jet.random,
- cern.jet.random.engine,
- edu.cornell.lassp.houle.RngPack,
- uk.ac.standrews.utils.main,
- uk.ac.standrews.utils.main.collectionutils,
- uk.ac.standrews.utils.main.collectionutils.setutils,
- uk.ac.standrews.utils.main.database,
- uk.ac.standrews.utils.main.diff,
- uk.ac.standrews.utils.main.guiutils,
- uk.ac.standrews.utils.main.maputils,
- uk.ac.standrews.utils.main.setutils,
- uk.ac.standrews.utils.main.stats,
- uk.ac.standrews.utils.main.stringutils,
- uk.ac.standrews.utils.main.webutils,
- uk.ac.standrews.utils.main.webutils.simpledomparser,
- uk.ac.standrews.utils.test,
- uk.ac.standrews.utils.test.database,
- uk.ac.standrews.utils.test.maputils,
- uk.ac.standrews.utils.test.setutils,
- uk.ac.standrews.utils.test.stats

## Class Hierarchy

- java.lang.Object
  - java.util.AbstractMap<K,V> (implements java.util.Map<K,V>)
    - java.util.TreeMap<K,V> (implements java.lang.Cloneable, java.util.NavigableMap<K,V>, java.io.Serializable)
      - uk.ac.standrews.utils.main.maputils.MapOfMaps
      - uk.ac.standrews.utils.main.maputils.OneToMany<T,U>
        - uk.ac.standrews.utils.main.maputils.OneToManyMap<T,U>
  - uk.ac.standrews.utils.test.AllDatabaseUtilsTests
  - uk.ac.standrews.utils.test.AllMapUtilsTests
  - uk.ac.standrews.utils.test.AllUtilsTests
  - uk.ac.standrews.utils.main.collectionutils.ArrayUtils
  - junit.framework.Assert
    - junit.framework.TestCase (implements junit.framework.Test)
      - uk.ac.standrews.utils.test.stats.AllStatsUtilsTests
      - uk.ac.standrews.utils.test.CollectionUtilsTest
      - uk.ac.standrews.utils.test.Data
      - uk.ac.standrews.utils.test.database.DbConnPropsTest
      - uk.ac.standrews.utils.test.database.DbCreatorTest
      - uk.ac.standrews.utils.test.database.DbManagerTest
      - uk.ac.standrews.utils.test.database.DbReaderTest
      - uk.ac.standrews.utils.test.database.DbUtilsTest
      - uk.ac.standrews.utils.test.database.DbWriterTest
      - uk.ac.standrews.utils.test.FileUtilsTest
      - uk.ac.standrews.utils.test.ListUtilsTest
      - uk.ac.standrews.utils.test.maputils.MapOfMapsTest
      - uk.ac.standrews.utils.test.maputils.MapUtilsTest
      - uk.ac.standrews.utils.test.NumberUtilsTest
      - uk.ac.standrews.utils.test.maputils.OneToManyMapTest
      - uk.ac.standrews.utils.test.stats.ParetoTest
      - uk.ac.standrews.utils.test.stats.PseudoTruncatedParetoTest
      - uk.ac.standrews.utils.test.stats.RandomUtilsTest
      - uk.ac.standrews.utils.test.ReflectionUtilsTest
      - uk.ac.standrews.utils.test.RegExpUtilsTest
      - uk.ac.standrews.utils.test.setutils.SetTest
      - uk.ac.standrews.utils.test.SimpleDOMParserTest
      - uk.ac.standrews.utils.test.database.SqlUtilsTest
      - uk.ac.standrews.utils.test.stats.StatUtilsTest
      - uk.ac.standrews.utils.test.StringUtilsTest
      - uk.ac.standrews.utils.test.setutils.SupersetsManagerTest
      - uk.ac.standrews.utils.test.database.TableCreatorTest
      - uk.ac.standrews.utils.test.maputils.TableMapTest
      - uk.ac.standrews.utils.test.database.TableReaderTest
      - uk.ac.standrews.utils.test.database.TableRowTest
      - uk.ac.standrews.utils.test.database.TableTest
      - uk.ac.standrews.utils.test.database.TableWriterTest
      - uk.ac.standrews.utils.test.TimeUtilsTest
      - uk.ac.standrews.utils.test.stats.TruncatedParetoTest
      - uk.ac.standrews.utils.test.stats.UniformRandomSingletonTest
      - uk.ac.standrews.utils.test.stats.UniformRandomUtilsTest
      - uk.ac.standrews.utils.test.UtilsTest
      - uk.ac.standrews.utils.test.WebUtilsTest
      - uk.ac.standrews.utils.test.XmlNodeTest
      - uk.ac.standrews.utils.test.XmlSearcherTest
      - uk.ac.standrews.utils.test.XmlUtilsTest
  - uk.ac.standrews.utils.main.webutils.ClientHttpRequestUtils
  - uk.ac.standrews.utils.main.collectionutils.CollectionUtils
  - uk.ac.standrews.utils.main.guiutils.CommandLineMenu
  - uk.ac.standrews.utils.main.guiutils.CommandOption
  - java.awt.Component (implements java.awt.image.ImageObserver, java.awt.MenuContainer, java.io.Serializable)
    - java.awt.Container
      - java.awt.Window (implements javax.accessibility.Accessible)
        - java.awt.Frame (implements java.awt.MenuContainer)
          - javax.swing.JFrame (implements javax.accessibility.Accessible, javax.swing.RootPaneContainer, javax.swing.WindowConstants)
            - uk.ac.standrews.utils.main.guiutils.GuiUtils
  - uk.ac.standrews.utils.main.database.DbConn (implements java.io.Serializable)
  - uk.ac.standrews.utils.main.database.DbConnectionSingleton
  - uk.ac.standrews.utils.main.database.DbManaged
    - uk.ac.standrews.utils.main.database.DbCreator
    - uk.ac.standrews.utils.main.database.DbReader
    - uk.ac.standrews.utils.main.database.DbWriter
  - uk.ac.standrews.utils.main.database.DbManagerSingleton
  - uk.ac.standrews.utils.main.database.DbUtils
  - uk.ac.standrews.utils.main.diff.Diff
  - uk.ac.standrews.utils.main.diff.Difference
  - uk.ac.standrews.utils.main.EntrezUtils
  - uk.ac.standrews.utils.main.FileUtils
  - uk.ac.standrews.utils.main.Initialised (implements uk.ac.standrews.utils.main.Initialisable)
  - uk.ac.standrews.utils.main.collectionutils.ListUtils
  - uk.ac.standrews.utils.main.LoggerCreator
  - uk.ac.standrews.utils.main.LogUtils
  - uk.ac.standrews.utils.main.database.Managed
  - uk.ac.standrews.utils.main.database.Manager
    - uk.ac.standrews.utils.main.database.DbManager (implements java.io.Serializable)
      - uk.ac.standrews.utils.main.database.TableManager
        - uk.ac.standrews.utils.main.database.TableCreator
        - uk.ac.standrews.utils.main.database.TableReader
        - uk.ac.standrews.utils.main.database.TableRow
        - uk.ac.standrews.utils.main.database.TableWriter
  - uk.ac.standrews.utils.main.maputils.MapUtils
  - uk.ac.standrews.utils.main.NumberUtils
  - uk.ac.standrews.utils.main.guiutils.OptionUtils
  - uk.ac.standrews.utils.main.PathUtils
  - cern.colt.PersistentObject (implements java.lang.Cloneable, java.io.Serializable)
    - cern.jet.random.AbstractDistribution (implements cern.colt.function.DoubleFunction, cern.colt.function.IntFunction)
      - cern.jet.random.AbstractContinousDistribution
        - cern.jet.random.Pareto
          - uk.ac.standrews.utils.main.stats.TruncatedPareto
            - uk.ac.standrews.utils.main.stats.PseudoTruncatedPareto
    - edu.cornell.lassp.houle.RngPack.RandomElement (implements java.lang.Cloneable)
      - edu.cornell.lassp.houle.RngPack.RandomJava
      - edu.cornell.lassp.houle.RngPack.RandomSeedable
        - cern.jet.random.engine.RandomEngine (implements cern.colt.function.DoubleFunction, cern.colt.function.IntFunction)
          - cern.jet.random.engine.MersenneTwister
        - edu.cornell.lassp.houle.RngPack.Ranecu
        - edu.cornell.lassp.houle.RngPack.Ranlux
        - edu.cornell.lassp.houle.RngPack.Ranmar
      - edu.cornell.lassp.houle.RngPack.RandomShuffle
  - uk.ac.standrews.utils.main.stringutils.Pool (implements java.io.Serializable)
    - uk.ac.standrews.utils.main.BooleanArrayPool
    - uk.ac.standrews.utils.main.stringutils.StringPool
  - uk.ac.standrews.utils.main.PropertiesUtils
  - uk.ac.standrews.utils.main.stats.PseudoTruncatedParetoSingleton
  - edu.cornell.lassp.houle.RngPack.RandomApp
  - uk.ac.standrews.utils.main.ReflectionUtils
  - uk.ac.standrews.utils.main.RegExpUtils
  - uk.ac.standrews.utils.main.database.ResultSetUtils
  - uk.ac.standrews.utils.main.Scripts
  - uk.ac.standrews.utils.main.setutils.Set (implements java.lang.Comparable<T>)
  - uk.ac.standrews.utils.main.collectionutils.setutils.Set (implements java.lang.Comparable<T>)
  - uk.ac.standrews.utils.main.setutils.SetUtils
  - uk.ac.standrews.utils.main.collectionutils.setutils.SetUtils
  - uk.ac.standrews.utils.main.webutils.simpledomparser.SimpleDOMParser
  - uk.ac.standrews.utils.main.guiutils.SimpleRadioButtonPanel
  - uk.ac.standrews.utils.main.database.SqlUtils
  - uk.ac.standrews.utils.main.stats.StatUtils
  - uk.ac.standrews.utils.main.stringutils.StringUtils
  - uk.ac.standrews.utils.main.setutils.SupersetsManager
  - uk.ac.standrews.utils.main.collectionutils.setutils.SupersetsManager
  - uk.ac.standrews.utils.main.SystemUtils
  - uk.ac.standrews.utils.main.database.Table
  - uk.ac.standrews.utils.main.database.TableColumn (implements java.lang.Comparable<T>)
  - uk.ac.standrews.utils.main.maputils.TableMap
  - uk.ac.standrews.utils.main.TimeUtils
  - uk.ac.standrews.utils.main.stats.UniformRandomSingleton
  - uk.ac.standrews.utils.main.stats.UniformRandomUtils
  - uk.ac.standrews.utils.main.Utils
  - uk.ac.standrews.utils.main.collectionutils.VectorUtils
  - uk.ac.standrews.utils.main.webutils.WebUtils
  - uk.ac.standrews.utils.main.webutils.simpledomparser.XmlNode
  - uk.ac.standrews.utils.main.webutils.simpledomparser.XmlSearcher
  - uk.ac.standrews.utils.main.webutils.XMLUtils

## Interface Hierarchy

- cern.colt.function.DoubleFunction
- uk.ac.standrews.utils.main.Initialisable
- cern.colt.function.IntFunction

- Overview
- Package
- Class
- Use
- Tree
- Deprecated
- Index
- Help

- Prev
- Next

- Frames
- No Frames

- All Classes
